# Influence of Technological Maturity on the Secondary Metabolites of Hemp Concentrate (*Cannabis sativa* L.)

**DOI:** 10.3390/foods10061418

**Published:** 2021-06-18

**Authors:** Bohuslava Tremlová, Hana Koudelková Mikulášková, Klaudia Hajduchová, Simona Jancikova, Dominika Kaczorová, Sanja Ćavar Zeljković, Dani Dordevic

**Affiliations:** 1 Department of Plant Origin Food Sciences, Faculty of Veterinary Hygiene and Ecology, University of Veterinary Sciences Brno, Palackého tř. 1946/1, 61242 Brno, Czech Republic; tremlovab@vfu.cz (B.T.); koudelkovamih@vfu.cz (H.K.M.); h17242@vfu.cz (K.H.); jancikovas@vfu.cz (S.J.); 2 Centre of the Region Haná for Biotechnological and Agricultural Research, Department of Genetic Resources for Vegetables, Medicinal and Special Plants, Crop Research Institute, Šlechtitelů 29, 78371 Olomouc, Czech Republic; dominika.kaczorova@upol.cz (D.K.); sanjacavar.sc@gmail.com (S.Ć.Z.); 3 Centre of Region Haná for Biotechnological and Agricultural Research, Czech Advanced Technology and Research Institute, Palacky University, Šlechtitelů 27, 78371 Olomouc, Czech Republic

**Keywords:** hemp, *Cannabis sativa* L., antioxidant activity, polyphenols, cannabinoids, cannabidiol, CBD, vegetation period

## Abstract

During the last decade, the popularity of hemp products has been rising rapidly. Products containing cannabidiol (CBD) are of predominant interest. Traditional hemp products are frequently enriched by CBD due to their potential therapeutic effects. Cannabidiol occurs naturally in hemp juice together with other biologically active substances, such as terpenes, flavonoids, and stilbenoids. These constituents act synergistically. This study aimed to observe the influence of the hemp plant developmental stage on its chemical composition and antioxidant activity. The hemp plants were analyzed during three vegetative stages, i.e., before, during, and after flowering. The collected samples were evaluated using the following analyses: total polyphenolic content and profile, terpenoid and cannabinoid contents, and ferric reducing antioxidant power. The results revealed statistically significant differences between the samples in almost all set parameters. The optimal period for hemp harvest depends on desirable compounds, i.e., phenolic content is the highest before flowering, while the levels of cannabinoids and terpenoids are the highest during the flowering period.

## 1. Introduction

Hemp (*Cannabis sativa* L.) is a dioecious, annual, and multipurpose crop. It is one of the oldest plants, and most likely originates from the Altai Mountains in Russia, from where it has spread globally [1]. Hemp plants are cultivated for the production of seeds, fiber, shives, or flowers, and they have to keep the EU legislative limit of psychoactive substance—0.2% of tetrahydrocannabinol (THC). These genotypes are often called fiber-type, and are used in industry and agronomy [2,3]. Up today, 75 hemp varieties allowed to be cultivated are registered into EU plant variety [4].

More than 550 compounds have been identified in cannabis [5]. Fatty acids, amino acids, and steroids are components of the plant’s primary metabolism, while components of its secondary metabolism include substances such as cannabinoids, terpenes, flavonoids, stilbenoids, and alkaloids. The content and proportion of these substances are influenced by several complexly related factors. The concentration of individual components depends mainly on the plant genotype, but also the physiological status and growing conditions [6].

The most controversial and discussed cannabis metabolites are cannabinoids [7]. Cannabinoids were originally referred to as a group of terpenoid substances unique to cannabis. They are specifically recognized by the endocannabinoid system in the human body. Cannabinoids synthesized by the plant are frequently called phytocannabinoids and the endogenous analogs endocannabinoids. Almost 150 phytocannabinoids have been reported to date [8].

Superfoods and novel foods have gained increased popularity in recent years. Hemp meal belongs to this type of food. It is rich in highly digestible proteins (29–31% of its content), and does not contain gluten. Thus, it is suitable for consumers with gluten intolerance [9]. The other products are extracts for the production of syrup and flavorings, hemp tea, or the herb itself [10]. Moreover, hemp products are used as food supplements with potential therapeutic effects.

In general, the main interest of hemp research is the content of cannabidiol (CBD), the non-psychoactive phytocannabinoid. Pre-clinical and clinical studies show potential therapeutic uses of CBD for several diseases [11]. The demand for this compound is rising, resulting in the expansion of different types of uses. Oils and tinctures are the most common CBD products. Cannabidiol can be isolated from extracts and added into food for the consumer’s attraction. Nevertheless, the increasing availability of CBD results in a discussion of the legislation, quality, and safety of this drug and novel food [12].

The present work is focused on the assessment of the appropriate period of harvest during plant vegetative and reproductive phases applicable to the production of hemp concentrate. It is comprised of an analysis of antioxidant activity, total phenolic content, phenolic profiling, and quantification of secondary metabolites (terpenes, phytocannabinoids). The samples were collected during three growing stages. The hemp concentrate, made of green plant upper parts, would be then used for the preparation of hemp juice.

## 2. Materials and Methods

### 2.1. Cultivation

**Figure 1:**
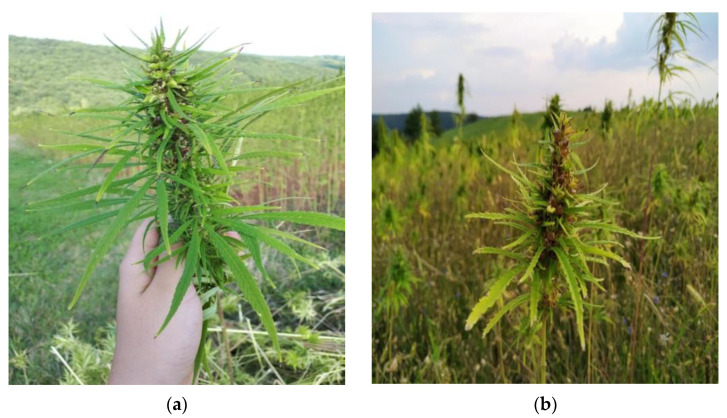
Investigated hemp plants (**a**) harvest 2018; (**b**) harvest 2019.

Hemp plants (cultivar USO-31) were cultivated for the production of hemp herb under non-standard agro-climatic conditions on clay-stony soil in the southern part of central Slovakia. The size of the managed soil was 0.42 hectares. No pre-crop was applied before sowing. In the autumn, classic manure was used as fertilizer, which was subsequently ploughed. In the spring, shallow ploughing was carried out. The seeds were sown by hand on 20 April 2019. Subsequently, the growing season began, which lasted until 12 September 2019, when it ended with the mowing of the stand using a drum rotary mower pulled by a tractor. During the growing season, the samples were taken three times, focusing on inflorescences and leaves. The first samples were harvested on 30 June 2019, before the flowering of the plant. The plants were short and reached a height of about 30 cm. The second samples were harvested on 3 August 2019 (the plants were already in the flowering phase). The inflorescences in the upper parts of the plant reached lengths of 2 to 30 cm. In this phase, there were also male inflorescences in the stand, which were already in a more advanced stage of flowering. The height of the plants ranged from 20–150 cm. The third sample was harvested on 1 September 2019, after flowering (Figure 1).

The production of the concentrate for the preparation of hemp juice itself consisted of drying green plant parts at a temperature of 145 °C for 25 min, and grinding the herb into a fine powder.

The samples of hemp (the same genus) after flowering harvested in 2018 were analyzed too. Hemp plants from the year 2018 season were much more developed, reaching a height of 2 m, and were more densely inseminated.

### 2.2. TPC (Total Polyphenols Content)

The total polyphenols were obtained after extraction of 1 g of the sample in water during 10 min of shaking. Then, 1 mL of the filtered extract was mixed with 5 mL of 1:10 Folin–Ciocalteu/water solution, as well as 4 mL Na_2_CO_3_ (75 g/L), and incubated in a 25 mL volumetric flask in the dark for 30 min. After incubation, the flask was filled to the designated mark, and the solution was measured at 765 nm on a spectrophotometer. The results are expressed as mg/g of gallic acid equivalent (GAE) because gallic acid was used to obtain the calibration curve [13]. The equation for the calibration curve was: y = 3.6574x + 0.0363; R^2^ = 0.9986.

### 2.3. Polyphenolic Profile (HPLC)

The HPLC 1260 Infinity (Agilent Technologies, Santa Clara, CA, USA) was used for the determination. The method by Gómez-Estaca et al. [14] was used, with some modifications. The 1% phosphoric acid (A) and acetonitrile (B) were used as a mobile phase as follows: 80% A and 20% B for 20 min, 70% A and 30% B for 20 to 25 min, 60% A and 40% B for 25 to 40 min. The separation was performed on Zorbax SB-C18 4.6 × 250 mm (Agilent Technologies, Santa Clara, CA, US. The flow rate was 1 mL/min, the injection volume was 10 μL, and the DAD setting was 324.5 nm. Each sample was analyzed in triplicate.

### 2.4. Terpenic Profile (GC-MS)

A representative sample of cannabis flowers was homogenized by mortar and pestle. Then, 100 mg of the homogenized sample was weighed in triplicates. The samples were extracted with 1.8 mL of 0.001% tridecane (internal standard) in hexane, and then sonicated for 30 min at room temperature and centrifuged 10 min (21.200× *g*, room temperature). The supernatant was injected into a gas chromatography Agilent 7890A GC coupled with a HP 5975C MSD spectrometer (Agilent Technologies, Inc., Santa Clara, CA, USA). Chromatographic separation was done on HP-5MS UI (30.0 × 250.0 μm; 0.25 μm film) (Agilent Technologies, Inc., Santa Clara, CA, USA). The temperature program was as follows: from 60 to 180 °C, increasing 3 °C/min (run time 40 min). Finally, the column was kept at 310 °C for 10 min at post run. The flow rate of helium was 1.1 mL/min, and the injection volume 1 μL. The temperatures of injection and detector were 250 and 230 °C, respectively. Quantification was done using an external calibration curve of standard mixtures of typical cannabis terpenes (Restek, Bellefonte, PA, USA).

### 2.5. Ferric Reducing Antioxidant Power (FRAP)

The FRAP method was implemented by the ultrasonic extraction of 0.1 g of the homogenized sample in 20 mL of 75% methanol, after which the 180 µL filtered extract was mixed with 300 µL of distilled water. The mixture was incubated with 3.6 mL of working solution (acetate buffer + TPTZ + FeCl_3_ × 6H_2_O in ratio 10:1:1) for 8 min in the dark, and then measured at 593 nm on a spectrophotometer CE7210 (Cecil Instruments, Cambridge, UK). The obtained results are expressed in µmol Trolox/g, since Trolox was used to make the calibration curve [15].

### 2.6. Cannabinoid Analysis

UHPLC-MS/MS analysis of cannabinoids was performed according to the previously reported methodology [16]. Briefly, 50 ± 1 mg of each sample was weighed in triplicates. The samples were extracted with 1.8 mL of 96% ethanol, and sonicated for 15 min at room temperature. After 10 min centrifugation (21.200× *g*, room temperature), the supernatant was diluted with 70% ACN (acetonitrile) with 0.1% formic acid. Internal standards (CBD-d_3_, CBN-d_3_, Δ^9^-THC-d_3_) were added at this step. The diluted samples were directly injected into an UltiMate™ 3000 UHPLC system (Thermo Fisher Scientific, Waltham, MA, USA). Chromatographic separation was done on a Luna Omega Polar C18 (100 × 2.1 mm; 1.6 μm particle size) UHPLC column (Phenomenex, Torrance, CA, USA) kept at 40 °C. The mobile phases were water (A) and acetonitrile (B), both containing 0.1% (*v/v*) formic acid. A binary gradient started at 60% B and was increasing to 80% B over the first 11 min. Then, an increase in B to 100% followed until 12.5 min. Finally, the column was re-equilibrated to the initial conditions for 4.5 min. The flow rate was 0.3 mL/min and the injection volume 2 μL. Neutral cannabinoids were detected in positive ionization mode ESI+, whereas negative ESI− was used for acidic ones.

### 2.7. Statistical Methods

Using SPSS software (version 23.0, SPSS, Chicago, IL, USA), the results were statistically evaluated based on a one-way analysis (ANOVA). Based on the homogeneity, variances determined *p* < 0.05 as a statistically significant difference. For values of *p* < 0.05, non-parametric Games–Howell tests were used. For *p* > 0.05, the parametric Tukey test was chosen.

## 3. Results and Discussion

Recently, interest in hemp cultivation has significantly increased, considering its positive impact on the environment and the production of feed and food of high nutritional value [17]. Research has been undertaken to assess the possibility of using hemp raw materials in products such as bread [18] and cookies [19]. The hemp proteins are characterized as a good protein source with adequate essential amino acids and excellent digestibility. The hydrolyzed peptides exhibit various health benefits, such as antioxidant activity, antihypertensive activity, and hypoglycemic activity [20].

The content of secondary metabolites is crucial for the quality evaluation of plant-based food samples. The content of cannabinoids, terpenes, polyphenolic compounds, their mutual interaction, and antioxidant activity are the observed parameters [21]. The present work is focused on the assessment of these compounds in the hemp concentrate, which was prepared from plants collected during the vegetative and reproductive phases.

### 3.1. Polyphenolic Compounds

The content of total polyphenol compounds in the analyzed hemp concentrate samples is shown in Table 1. Statistically (*p* < 0.05), the sample before flowering had the highest amount of total polyphenol content (Sample A: 17.22 ± 0.01 mg/g), while the sample after flowering had the lowest content (Sample C: 6.62 ± 0.01 mg/g).

The values of the total polyphenolic content differed in all samples with a statistical significance (*p* < 0.01). The highest content of polyphenols was determined in the sample before flowering (A) 17.22 ± 0.01 mg/g. Almost three times fewer compounds were detected in the sample collected during flowering (B), and the sample after flowering (C) contained the least amount of the compounds (6.62 ± 0.01 mg/g). It might be concluded that the content of polyphenols decreased as the plants aged. These results are consistent with the literature [6,22]. Moreover, the levels of flavonoids are normally higher in cannabis leaves than in inflorescences [23]. Sample A was predominantly composed of leaves before flowering, and hence it had the highest polyphenolic content. During the reproductive phase, the plant concentrates more on flower generation than on leave multiplication.

The phenolic compounds contribute to the antioxidant capacity of plant material [24,25]. During the analysis, the decrease in concentration of polyphenolic compounds during the vegetative stage of hemp plants was observed. There was a difference in the polyphenol content in samples C and D, both collected after flowering but in the different year seasons. The higher content was recorded for sample D from 2018. This could be explained by different environmental factors that highly influence the secondary metabolism of plants. Although the plants were grown under the same agro-technical conditions, the climatic conditions were not the same in these two years. According to data from the Slovak Hydrometeorological Institute, 2019 had much less precipitation than 2018, especially during the summer period [26,27]. The values of analyzed samples were significantly lower compared to the results reported earlier [28]. The concentration of gallic acid in the cannabis sample was 29.98 ± 0.56 mg/g in the compared study [28]. The dissimilar quality of plants might be due to the overall weakness of the tested plants from a morphological and phytochemical point of view. The difference in polyphenolic compounds could be caused by different types of cannabis plants. Cannabis is a highly diverse plant, and the content and metabolite profile differ between varieties [29]. In the study, the plants of species indica were tested, which could show a greater content of phenolic compounds.

The results were also compared to young green barley [30]. The analyzed product was made from the green plant parts, and was processed similar to the present study. The highest values ranged from 4.962 to 5.916 g/kg of ferulic acid. The observation of a decrease in polyphenolic compounds during plant aging was confirmed in both studies.

**Figure 2:**
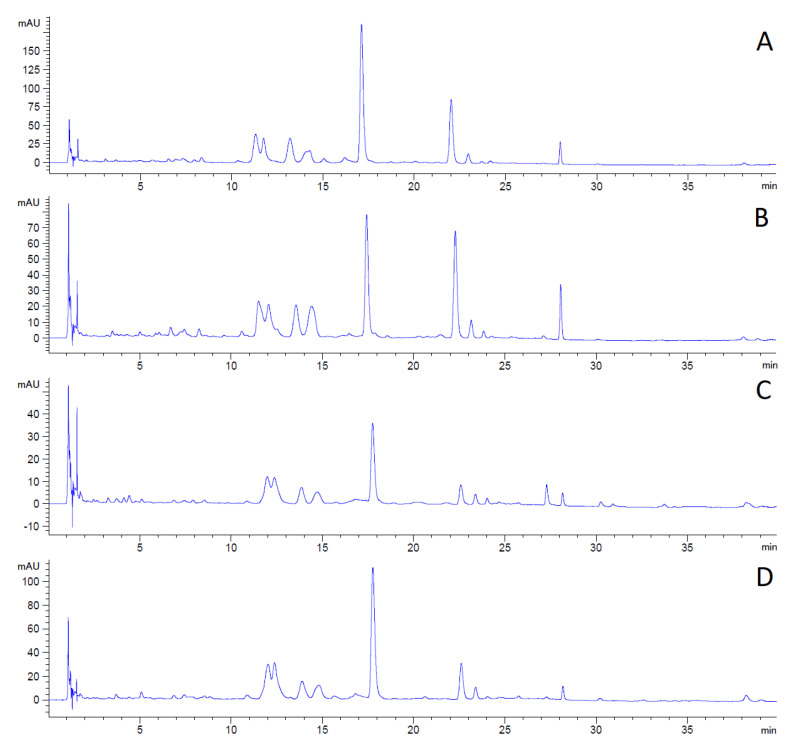
LC chromatograms of the hemp concentrates, measured at 270 nm. (**A**)—before flowering 2019, (**B**)—flowering 2019, (**C**)—after flowering 2019, (**D**)—after flowering 2018.

The changes in the content and composition of polyphenolic compounds during plant growth can be observed in the following LC chromatograms (Figure 2).

The first chromatogram (Figure 2A) shows the polyphenolic profile of hemp concentrate from the plants collected before flowering in 2019. Sample A contained the highest amount of phenolics as the peaks reached the highest intensity of the analyzed samples.

The decline in the concentration of all detected compounds is apparent in sample B (Figure 2B) in comparison with sample A. The decrease in intensity by 56% for the analyte eluted in 17 to 18 min is detected in the second chromatogram (Figure 2B).

The third chromatogram (Figure 2C) shows a continuing decrease in phenolic compounds in sample C. Some of the compounds detected in samples A and B are absent in sample C. The total compound decline is 80% compared to sample A.

The phenolic assay confirmed that the content of polyphenolic compounds decreased during plant aging.

The last chromatogram (Figure 2D) shows the content of polyphenolics in sample D, collected after flowering in 2018. The analysis differs significantly from the hemp concentrate from the same vegetative stage but the following year (Sample C). The content of compounds was higher in sample D (by 225%). On the other hand, the content was 33% lower in sample D than in sample A. According to the obtained results, it could be concluded that the plant is highly sensitive to cultivation conditions. These factors may have a greater effect on the content of metabolites than the vegetative plant stage during the time of harvest. Furthermore, the hemp concentrate preserved high amounts of phenolics even though it was collected the previous year and stored. It may be concluded that the phenolics compounds are stable in stored plant material.

### 3.2. Terpenic Profile

**Figure 3:**
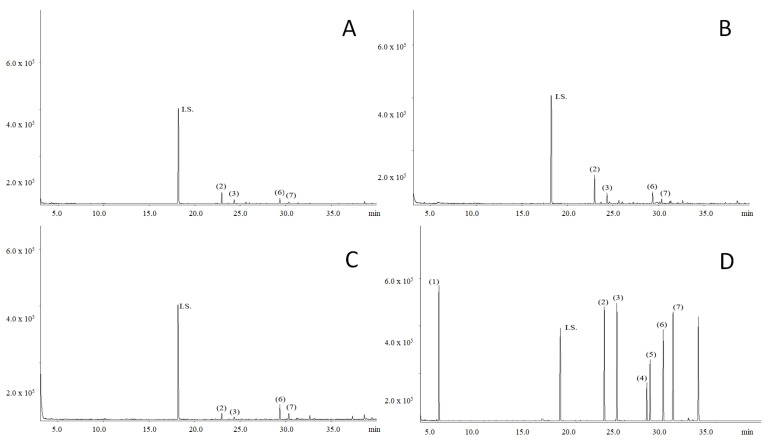
Terpenoid profiles of the investigated hemp samples. I.S.—*n*-tridecane, 1—α-pinene, 2—β-caryophyllene, 3—α-humulene, 4—*Z*-nerolidol, 5—*E*-nerolidol, 6—caryophyllene oxide, 7—guaiol, (**A**)—before flowering 2019, (**B**)—flowering 2019, (**C**)—after flowering 2019, (**D**)—after flowering 2018.

Table 2 represents the content of terpenes (μg/g) in hemp concentrates, while their profiles are shown in Figure 3. The values of β-caryophyllene were the highest during the flowering season, which is in agreement with the literature [31]. After flowering, the content of β-caryophyllene is decreasing rapidly. Moreover, a similar trend was observed for α-humulene. The terpenoid contents differ between samples with statistical significance (*p* < 0.05). Similarly, as in the previous analysis, sample D contained the highest amounts of terpenes. Furthermore, its profile was different; more terpenes were detected and quantified in this sample (α-pinene, Z-nerolidol, E-nerolidol). A statistically significant increase in β-caryophyllene and α-humulene contents were detected in samples that were collected during flowering (B). On the other hand, a statistically significant decrease in caryophyllene oxide and guaiol contents were detected for collected samples before flowering.

### 3.3. Cannabinoids

Cannabinoids have a neuroprotective effect, hence they can be used in the treatment of neurodegenerative diseases or epilepsy. In natural medicine, it is also used as an analgesic and anti-inflammatory medicine [32].

The results of the quantification of phytocannabinoids in hemp concentrates are presented in Table 3. The total cannabinoid content was lower by 60% in the samples from 2019 than in the sample from the year 2018. The content rose by 70% between the sampling before and during flowering, and the difference in content during and after flowering was 5%. It could be assumed that the total cannabinoid content is primarily influenced by environmental conditions during cultivation. Cosentino et al. (2012) stated that sowing in the middle and at the end of April could shorten the flowering period and cause the presence of short stems and generally lower yields of seeds and fiber [33]. As a result, the plants may grow during shorter days and the photoperiod is shortened. Thus, it significantly affects plant development in the initial phase of growth. The sowing occurred on April 20 in 2019, and the yield was probably lower due to the deficiency of sunlight hours and unfavorable weather.

The content of the major hemp cannabinoid cannabidiol (CBD) is not constant during plant life. The content strongly increases during flowering, and falls after flowering [34,35]. The cannabinoid assay confirmed this status. The content of CBD was higher in the sample from harvest in 2018 than in that of the following year. The study of Rustichelli et al. (1998) focused on the measurement of CBD content in hemp varieties Carmagnola, Fibranova, and Ungherese [36]. The plant flowers were sampled during different phases of maturation in the months June, July, August, and September as in the present study. In both studies, the highest amount of CBD was measured in the flowering stage.

The acidic form of cannabidiol—cannabidiolic acid (CBDA)—was also assayed in hemp concentrate. This compound was particularly present before the heat treatment, which then activated the decarboxylation of the acid to the neutral form. The highest amount of CBDA (7.78 ± 0.36 mg/g) was found in sample D, collected after flowering in 2018. The linear increase in CBDA concentration during the vegetative stage could be stated from the results of harvest 2019.

The sum of CBD concentration was significantly different between the samples after flowering. Surprisingly, the ratio of CBD to CBDA differed between samples C and D. CBDA was dominant in sample D. Conversely, sample C contained more CBD than the parallel acid. Sample D comprised of 1.13% of total CBD (CBD + 0.877 *CBDA), and this concentration exceeded the conventional content characteristic for this variety. The general content of CBD in the variety USO 31 ranges from 0.5 to 1% [37,38].

An increased amount of cannabigerol (CBG) was observed during the flowering stage. Thus, cannabinoids have the highest concentration in this growth stage, which corresponds with previously published data The concentration of CBG is generally low and might be under the limit of quantification [39].

The limit for THC content in hemp is set at 0.2% in European Union (Regulation (EU) No 1307/2013 of the European Parliament and of the Council). The samples did not exceed this limit. The highest amount of total THC was found in sample D (0.06%). In the samples from 2019, tetrahydrocannabinolic acid (THCA) was not quantified due to the low concentration under the limit of quantification.

### 3.4. Antioxidant Capacity

The antioxidant capacity evaluated by FRAP method is shown in Table 4.

From Table 4, the decrease in antioxidant capacity (*p* < 0.05) with the increasing technological maturity can be observed within obtained samples from the same growing season (A, B, and C). The average value of A (the sample taken before flowering) was 0.246 ± 0.003 µmol (Trolox)/g, similar to the result for the USO hemp variety 0.201 ± 0.003 µmol (Trolox)/g in Nagytė et al. (2018) [40]. The decrease in antioxidant capacity could be caused by the aging of the plant and the subsequent decomposition of secondary metabolites with antioxidant potential [41].

**Figure 4:**
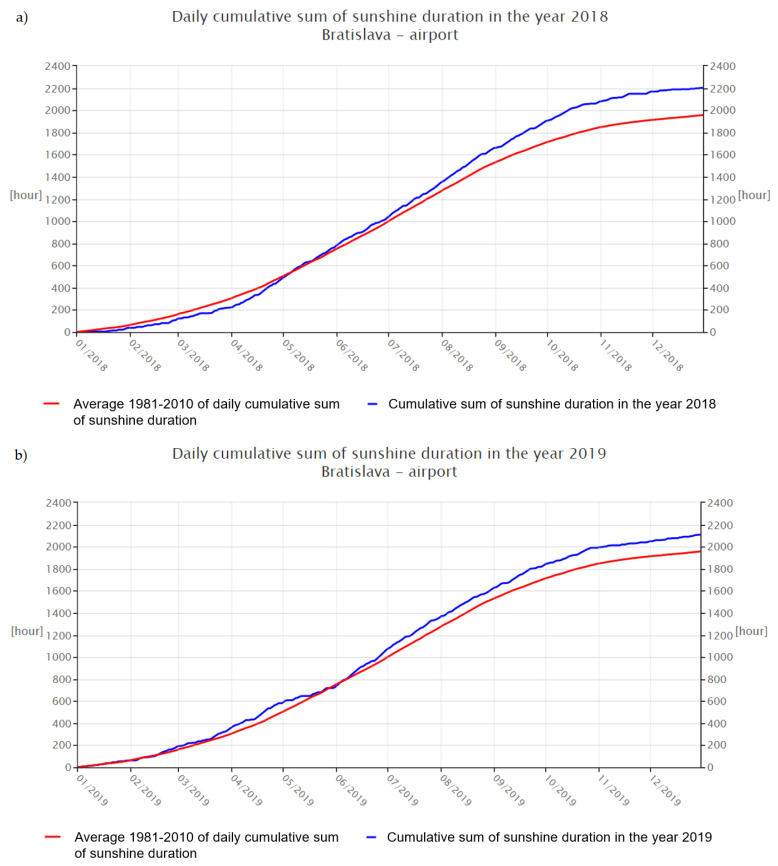
The graphs of sunny hours: (**a**) sunshine 2018 [26], (**b**) sunshine 2019 [27].

**Figure 5:**
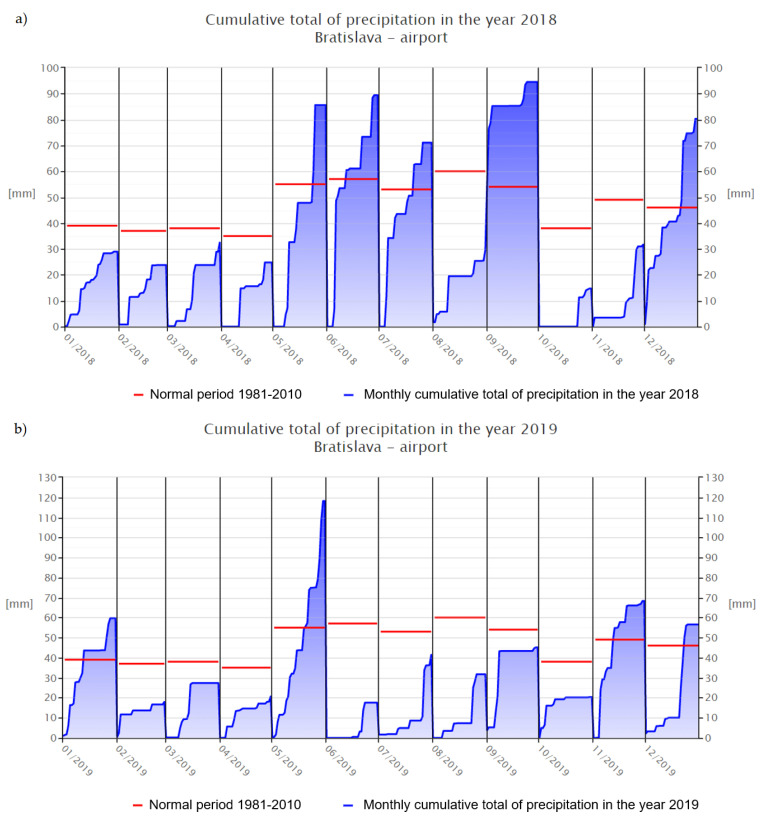
The graphs of precipitation: (**a**) 2018 [42], (**b**) 2019 [43].

The antioxidant capacity of the sample from 2018 (D) was significantly (*p* < 0.05) higher than sample C, since they both were taken in the same growing season. Low antioxidant activity of C in comparison with the collection obtained from the previous year (D) could be caused by unfavorable environmental conditions that had a negative impact not only on the overall morphological appearance of the plants, but also on their phytochemical profiles. The main factors that could confirm the following statement are the number of sunny hours during growing seasons 2018 (1331 h) and 2019 (1237 h), or precipitation (Figure 4 and Figure 5) [26,27,42,43]. The study of Sikora et al. (2011) states information about the influence of agro-climatic conditions on the content of main cannabinoids in *Cannabis sativa* L., that precipitation has a negative influence on the CBD content of industrial hemp [44]. It is the length and intensity of illumination that play the most important role in the formation of polyphenols, which have an antioxidant character [45].

The antioxidant capacity of hemp extracts was evaluated before, and values ranged from 45.9 ± 0.4 to 63.6 ± 0.9 µmol (Trolox)/g [46]. The antioxidant capacity of the extracts was higher than the measured value of hemp concentrate. The reason might be a higher concentration of secondary metabolites in the extract, whose preparation is more complex than the homogenization and heat treatment of a hemp concentrate sample. The drying process is suited for sample preparation due to the preservation of bioactive constituents in the sample [47].

## 4. Conclusions

Products containing hemp are becoming increasingly popular, and their demand is expanding. However, the market with these products is a new and unexplored area. It brings unclarified questions regarding the problematics of legislation, quality, and safety of these products. The research is frequently focused on the secondary metabolites, which may have health-promoting effects. They eventually have supportive properties in the treatment of numerous disorders. These compounds act as natural antioxidants and could be used in the preparation of food and supplements with preventive and therapeutic effects related to oxidative stress.

The highest content of polyphenolic compounds was analyzed in the sample harvested before flowering (17.217 ± 0.01 mg/g). This finding was following the highest values of antioxidant activity (0.246 ± 0.003 µmol TE/g) since it was observed in the same sample. According to this study, the harvest in June (before flowering) is the most convenient for the achievement of the highest antioxidant activity and polyphenolic content. Among the secondary metabolites in hemp, terpenes and cannabinoids are the most attractive for research and further processing. The highest amounts were found in the sample collected during flowering.

The concentrations of major terpenes β-caryophyllene and α-humulene were maximal during flowering, at 72.10 ± 0.42 and 36.75 ± 0.54 mg/g respectively. The accumulation of cannabinoids was also highest at this stage. The concentration of cannabidiol was 3.81 ± 0.09 mg/g. The harvest of female plants is essential in July due to the CBD concentration maximum.

The results of this study indicate that the environmental conditions during the vegetative stage are a significant factor in influencing the concentrations of secondary metabolites. The study underlined the seasonal variability as the important factor that can produce differences in the fingerprints in the same year and especially in different years.

## Figures and Tables

**Table 1:** The total content of polyphenolic compounds in hemp concentrates.

Sample	Polyphenol Content (mg/g of Gallic Acid)
A-before flowering 2019	17.22 ± 0.01 ^a^
B-flowering 2019	11.15 ± 0.01 ^b^
C-after flowering 2019	6.62 ± 0.01 ^c^
D-after flowering 2018	16.13 ± 0.003 ^d^

Letters a–d mark statistically significant difference between the samples (*p* < 0.05).

**Table 2:** Terpenes content in samples of (*Cannabis sativa* L.) concentrates.

Terpenes Content (µg/gDW)				
Terpene	A *	B	C	D
α-pinene	-	-	-	18.84 ± 1.06
β-caryophyllene	46.05 ± 0.90 ^a^	72.10 ± 0.42 ^b^	30.14 ± 1.10 ^c^	385.59d ± 6.14
α-humulene	27.38 ± 0.41 ^a^	36.75 ± 0.54 ^b^	22.79 ± 0.65 ^c^	129.39d ± 1.88
*Z*-nerolidol	-	-	-	93.58d ± 3.63
*E*-nerolidol	-	-	-	63.90d ± 1.54
Caryophyllene oxide	41.77 ± 0.81 ^a^	55.58 ± 0.93 ^b^	58.73 ± 1.34 ^c^	125.72d ± 2.15
Guaiol	23.82 ± 0.46 ^a^	28.04 ± 0.43 ^b^	30.91 ± 1.00 ^c^	50.38d ± 1.94

Small letters (a, b, c) in the upper index mark statistically significant difference (*p* < 0.05) between the lines. * A—before flowering 2019, B—flowering 2019, C—after flowering 2019, D—after flowering 2018.

**Table 3:** CBD and THCA contents in hemp concentrates (mg/gDW).

	A *	B	C	D
CBDV	0.00 ± 0.00	0.024 ± 0.00	0.014 ± 0.001	0.022 ± 0.001
CBDA	0.26 ± 0.02 ^a^	1.11 ± 0.05 ^b^	2.03 ± 0.39 ^b^	7.78 ± 0.36 ^c^
CBG	0.041 ± 0.003 ^a^	0.250 ± 0.011 ^b^	0.033 ± 0.005 ^a^	0.081 ± 0.003 ^c^
CBD	1.187 ± 0.064	3.838 ± 0.093	2.893 ± 0.030	4.453 ± 0.160
CBN	0.005 ± 0.001 ^a^	0.013 ± 0.002 ^a^	0.014 ± 0.001 ^a^	0.028 ± 0.001 ^b^
Ʃ	1.493	5.235	4.984	12.364
Δ^9^-THC	0.032 ± 0.002	0.111 ± 0.003	0.094 ± 0.006	0.365 ± 0.014
THCA	0.00 ± 0.00	0.00 ± 0.00	0.00 ± 0.00	0.250 ± 0.025

Small letters (a, b, c) in the upper index mark statistically significant difference between the samples (*p* < 0.05). * A—before flowering 2019, B—flowering 2019, C—after flowering 2019, D—after flowering 2018.

**Table 4:** Antioxidant capacity of hemp concentrate.

Samples	FRAP (µmol TE/g)
A—before flowering 2019	0.246 ± 0.003 ^a^
B—flowering 2019	0.117 ± 0.002 ^b^
C—after flowering 2019	0.073 ± 0.001 ^c^
D—after flowering 2018	0.216 ± 0.002 ^d^

Small letters in upper index mark statistically significant difference between the samples (*p* < 0.05). TE—Trolox equivalents. A—before flowering 2019, B—flowering 2019, C—after flowering 2019, D—after flowering 2018.
